# Assessing mental health, cognitive function and quality of life of breast cancer patients: exploring associations with gut microbiota in an observational and preliminary study

**DOI:** 10.3389/fpsyg.2026.1437697

**Published:** 2026-03-11

**Authors:** Catarina Calafate, Diogo Alpuim Costa, Teresa Campos, Pedro Casal Ribeiro, Filipa Martinho, Cristina Freitas, Carolina Botelho de Sousa, Ida Negreiros, Ana Canastra, Paula Borralho, Ana Guia Pereira, Cristina Marçal, Rita Ribeiro, José Germano Sousa, Catarina Dinis, Renata Chaleira, Rita Calha, Júlio César Rocha, Conceição Calhau, André Moreira-Rosário, Ana Faria

**Affiliations:** 1 Faculdade de Ciências Médicas (FCM), NOVA Medical School (NMS), Universidade NOVA de Lisboa (UNL), Lisbon, Portugal; 2 Haematology and Oncology Department, CUF Oncologia, Lisbon, Portugal; 3 Medical Oncology Department, Hospital de Cascais, Cascais, Portugal; 4 Breast Unit, CUF Oncologia, Lisbon, Portugal; 5 Centro de Medicina Laboratorial Germano de Sousa, Lisbon, Portugal; 6 Anatomic Pathology Department, CUF Oncologia, Lisbon, Portugal; 7 Faculdade de Medicina da Universidade de Lisboa (FMUL), Lisbon, Portugal; 8 Psychology Department, CUF Cascais Hospital, Cascais, Portugal; 9 Psychology Department, CUF Descobertas Hospital, Lisbon, Portugal; 10 Nutrition Department, CUF Cascais Hospital, Cascais, Portugal; 11 Faculdade de Ciências Médicas (FCM), NOVA Medical School (NMS), CINTESIS@RISE, Universidade NOVA de Lisboa (UNL), Lisbon, Portugal; 12 Unidade Universitária Lifestyle Medicina José de Mello Saúde, NOVA Medical School, Lisbon, Portugal; 13 Faculdade de Ciências Médicas, CHRC - Comprehensive Health Research Center, NOVA Medical School, Lisbon, Portugal

**Keywords:** anxiety, breast cancer, cognitive function, depression, microbiota, microbiome, quality-of-life

## Abstract

**Introduction:**

Breast cancer patients face several physical and psychological problems, such as anxiety, depression, and cognitive dysfunction. The disease and treatments can also impact the microbiota, which is associated with cognitive and psychological issues and, consequently, affected quality-of-life (QoL). This study aimed to correlate the initial gut microbiota of newly diagnosed HR+ (Hormone Receptor)/HER2- breast cancer patients with their mental health, cognitive function, and QoL at baseline and after 3 months of neoadjuvant chemotherapy.

**Materials and methods:**

This is a prospective, longitudinal, observational, exploratory study. Newly diagnosed HR+/HER2- breast cancer patients undergoing neoadjuvant chemotherapy were recruited upon diagnosis. At baseline (before neoadjuvant chemotherapy), general and lifestyle information, adherence to the Mediterranean diet, biochemical analysis, gut microbiota profile, the European Organization for Research and Treatment of Cancer Quality-of-Life Questionnaire Core-30 (EORTC QLQ-C30), the Montreal Cognitive Assessment (MoCA) and the Hospital Anxiety and Depression Scale (HADS), were collected. The EORTC QLQ-C30, MoCA, and HADS were repeated 3 months later.

**Results:**

From the 11 participants, most showed mild cognitive impairment at baseline, and there was no clear trend of improvement or deterioration at 3 months. Participants had borderline anxiety at baseline, which improved to a normal range, while depression remained stable. QoL declined for most women, with over 70% experiencing problems at 3 months. The association of these parameters with microbiota profile suggested that women with poorer cognitive function over time had lower Shannon index and microbial richness. Women with improved scores in the depression subscale of the HADS appear to have higher Shannon index and lower richness. Contrarily, Shannon index was lower and richness was higher for improved anxiety and global QoL scores. The results also suggest that changes in the abundance of various *genera* and *phyla* may be linked to the evolution of scores for the 3 questionnaires.

**Conclusion:**

Our study suggests a link between the microbiota profile at diagnosis and the psychological symptoms that develop at 3 months of breast cancer treatment. These findings shed light on potential strategies for positively modulating the microbiota to help enhance the body's resilience, particularly mental health, throughout the disease and treatments.

## 1 Introduction

The collection of bacteria, viruses, fungi, protozoa, and archaea that colonize the human body is referred to as the microbiota (Sampsell et al., 2020; Fernández et al., 2018; Shreiner et al., 2015). Various factors can shape the microbiota's composition, which suffers some transitional changes through life (Fernández et al., 2018; Dong and Gupta, 2019; Jeffery and Toole, 2013; Singh et al., 2017; Alpuim Costa et al., 2021). As a result, the human microbiota is a dynamic organ that participates in the immune system maturation and defense against pathogens, directly or indirectly affecting most of the host's physiologic functions (Fernández et al., 2018; Shreiner et al., 2015; Noce et al., 2019). Regarding the microbiotas' metabolic functions, it is involved in the breakdown of complex carbohydrates (such as indigestible dietary fibers) and the generation of beneficial metabolites, such as short-chain fatty acids (SCFA), which have a critical role in health and disease, with an impact in the modulation of immune responses and tumorigenesis (Shreiner et al., 2015; Jeffery and Toole, 2013; Singh et al., 2017).

A symbiotic relationship between the microbiota and the host is crucial for health outcomes. A bidirectional communication system between the central nervous system (CNS) and enteric nervous system exists, the so-called microbiota-gut-brain axis, making the human brain very sensitive to changes in the microbiota, which can potentiate the onset of anxiety, depression, and altered cognitive function (Ciernikova et al., 2021; Subramaniam et al., 2020; Du et al., 2020).

The gut microbiota, an essential modulator of the gut–brain axis interaction (Ciernikova et al., 2021; Subramaniam et al., 2020; Du et al., 2020), can affect the synthesis of the 5-hydroxytryptamine (5-HT), a precursor of serotonin, via immunological, neuroendocrine and direct neural mechanisms, as well as control tryptophan and gamma-aminobutyric acid (GABA) metabolism in the gut. Additionally, it has the ability to secrete and upregulate metabolites, such as SCFAs and brain-derived neurotrophic factor (BDNF; Du et al., 2020; Simpson et al., 2021). The microbiota can also play a role in the development and function of the hypothalamic-pituitary-adrenal (HPA) axis. In a state of anxiety and depression, there can be a dysregulated HPA signaling associated with higher levels of inflammatory mediators and cortisol and, consequently, a proinflammatory state. On the other hand, this inflammatory state can also lead to dysbiosis, increasing the intestinal permeability and translocation of lipopolysaccharides (LPS) into the bloodstream, potentiating CNS inflammation even further. This association between peripheral inflammation and brain function occurs via signaling across the blood-brain-barrier (BBB) and permeation of immune cells into the brain, which can contribute to a state of anxiety and depression (Simpson et al., 2021). This inflammatory state can also contribute to the activation of microglia and astrocytes and the consequent release of more pro-inflammatory cytokines and reactive oxygen species, potentiating the decline of cognitive function (Ciernikova et al., 2021; Subramaniam et al., 2020; Rodríguez Martín et al., 2020; Bajic et al., 2018). The role of microorganisms in inflammation is not only associated with the increase of species that potentiate inflammation, but also with the decrease of anti-inflammatory ones, such as the reduction in SCFA-producing species (Du et al., 2020; Simpson et al., 2021; Chang et al., 2022).

The role of microbiota in pathologies other than the obvious gastrointestinal has come to the researchers' attention, and indeed, evidence has shown that microbiota can also have a role in breast cancer. Compared to healthy individuals, many breast cancer patients present dysbiosis, with more pathogenic microorganisms and less Shannon index (Sampsell et al., 2020; Fernández et al., 2018; Yang et al., 2017). As previously mentioned, dysbiosis can lead to spontaneous inflammation of the mucosal barrier and greatly impact the host's health, with the possible onset of many diseases, including breast cancer. Therefore, a dysbiotic state may eventually potentiate oncogenesis, tumor progression and affect the response to treatments and their toxicity (Fernández et al., 2018; Singh et al., 2017; Alpuim Costa et al., 2021; Noce et al., 2019; Yang et al., 2017).

Breast cancer occurs when there is an uncontrollable growth of cancerous cells (Veronesi et al., 2005; American Cancer Society, 2021; Ahmad, 2021; National Cancer Institute, 2021). It is a complex disease with a still unknown etiology, involving a combination of genetic, epigenetic and environmental factors (Fernández et al., 2018; Winters et al., 2017; World Health Organization, 2021). The risk factors for breast cancer are most often associated with environmental, reproductive, and lifestyle factors, with less than 10% being attributed to an inherited genetic mutation. The gut microbiota has also been increasingly associated with breast cancer through its direct and indirect interference in various processes, such as immune system function, cell proliferation, and death, chronic inflammation, oncogenic signaling, and hormonal pathways (Alpuim Costa et al., 2021; Veronesi et al., 2005; Winters et al., 2017; Cardoso et al., 2019; World Health Organization, 2025; Rojas and Stuckey, 2016; Coughlin, 2019).

Several pro-carcinogenic mechanisms involve the microbiota (Fernández et al., 2018; Alpuim Costa et al., 2021; Yang et al., 2017; Villéger et al., 2019). The dysbiosis-related mechanisms described above for the onset of anxiety, depression, and altered cognitive function are also the ones present in the increased breast cancer risk (Sampsell et al., 2020; Ciernikova et al., 2021; Bajic et al., 2018). Breast cancer patients face several physical and psychological problems, including the physical symptoms of cancer itself and side effects from the treatment, such as loss of lean body mass, anxiety, depression, fatigue, pain, and altered neurocognitive function (Sampsell et al., 2020; Ciernikova et al., 2021; Bajic et al., 2018). Evidence shows that up to 50% of breast cancer patients have a diagnosis of anxiety, depression, or both in the year after diagnosis. Adjuvant chemotherapy might increase the risk of anxiety, depression, or both during but not after treatment. The estimated prevalence is highest during the acute phase of cancer treatment, which is particularly relevant since there is an association between depression and poor adherence to treatment and survival (Pitman et al., 2018; Smith, 2015; Maitiniyazi et al., 2022; Bower, 2008; Li et al., 2015). The microbiota, apart from being associated with cancer, can also interact with anti-cancer treatments. The gut microbiota can influence chemotherapy's response and side effects through its impact on drug pharmacokinetics, immune response modulation, or effect on local inflammation and gut barrier function (Sampsell et al., 2020; Alpuim Costa et al., 2021; Ciernikova et al., 2021; Bajic et al., 2018; Villéger et al., 2019; Roy and Trinchieri, 2017).

In addition to anxiety and depression, cancer-related cognitive impairment occurs in 15 to 45% of patients undergoing anti-cancer therapy (Bajic et al., 2018). Especially in the context of breast cancer, over half of patients report cognitive complaints after chemotherapy (Lange et al., 2019). Due to the non-selective nature of chemotherapy, it also targets healthy cells, leading to several adverse effects (Subramaniam et al., 2020; Bajic et al., 2018). Notably, it can induce neurotoxicity, leading to many patients complaining of altered cognitive function, such as impaired memory, attention, concentration, word-finding, processing speed, reaction time, executive functioning, and others (Ciernikova et al., 2021; Bajic et al., 2018; Lange et al., 2019; Hermelink, 2015). These cognitive challenges have broader implications, affecting patients' self-esteem, relationships, employment, finances, and independence. Moreover, patients with more significant cognitive impairment presented more severe levels of anxiety and depression (Ciernikova et al., 2021; Rodríguez Martín et al., 2020; Lange et al., 2019; Hermelink, 2015). While cognitive impairment may be present before treatment initiation, potentially linked to personal or environmental variables, such as the emotional state, it typically intensifies after chemotherapy (Rodríguez Martín et al., 2020).

In addition to their impact on mental health and cognition, breast cancer and the treatments have the potential to alter the microbiota, contributing to the onset of anxiety, depression, and altered cognitive function. These intricate challenges associated with breast cancer and its treatments could affect all quality-of-life (QoL) domains (Lopes-Conceição et al., 2021). QoL is intricately linked to psychosocial distress experienced by patients, including depression, anxiety, body image problems, fear of recurrence and death, and physical symptoms, including pain, fatigue, and sleep disturbance. Sociodemographic, clinical, and treatment factors, such as age, education, cancer stage, chemotherapy, and surgery, also play pivotal roles in shaping QoL (Smith, 2015; Bower, 2008; Lopes-Conceição et al., 2021; Mokhtari-Hessari and Montazeri, 2020; Park et al., 2021).

It is crucial to note that some of the symptoms of anxiety and depression may be misconstrued as side effects from cancer or treatments, leading clinicians to dismiss them. This oversight denies patients proper management, further contributing to a compromised QoL (Smith, 2015; Park et al., 2021; Iconomou et al., 2004). This aspect gains heightened significance considering the observed association between QoL and continuity of care, adherence to medical advice, and even length of survival (Smith, 2015; Park et al., 2021; Iconomou et al., 2004). Despite survival rates exceeding 90% for breast cancer patients, women with breast cancer experience the highest disability-adjusted life years lost among all types. Consequently, finding strategies to address this issue becomes imperative (Ciernikova et al., 2021; World Health Organization, 2021, 2020).

The main goal of the present study was to correlate the initial gut microbiota (before neoadjuvant chemotherapy) of newly diagnosed HR+/HER2- breast cancer patients with their mental health, cognitive function, and QoL at baseline and after 3 months of chemotherapy.

## 2 Materials and methods

The study protocol was approved by the Ethics Committee of CUF (Ref. 152/CE/JMSis) and NOVA Medical School (Ref. 166/2022/CEFCM). The study was conducted in accordance with the ethical principles expressed in the Declaration of Helsinki, Portuguese law, and Good Clinical Practice guidelines.

### 2.1 Study design

The “BioBreast Brain Study” is a prospective, longitudinal, observational, exploratory study. The participants were newly diagnosed HR+/HER2- breast cancer patients undergoing neoadjuvant chemotherapy at CUF *Oncologia*. The chemotherapy regimen consisted of 4 cycles of a combination of cyclophosphamide with doxorubicin, administered every 2 (dose-dense) or 3 weeks, followed by 12 cycles of weekly paclitaxel. Given the constraints of this preliminary analysis, it is crucial to highlight that, during the assessment, patients had not yet finished the entire 12 cycles of paclitaxel.

### 2.2 Participants recruitment

The sample was selected based on eligibility criteria, by convenience, upon diagnosis of breast cancer at the multidisciplinary tumor board. This was an exploratory study, and the sample size resulted from the eligible patients and recruitment viability for 6 months.

All clinical samples were obtained from subjects who provided written informed consent. According to the General Data Protection Regulation, the investigators ensured the subject's privacy and data confidentiality.

The participants recruited were women of at least 18 years of age who had a primary diagnosis of invasive breast cancer, HR+ (estrogen receptor and/or progesterone receptor ≥ 1% by IHC [Immunohistochemistry]) and HER2- (IHC 0/2+ and/or FISH (fluorescence *in situ* hybridization)/CISH (Chromogenic *in situ* hybridization) negative) according to local assessment, within II-IIIC breast cancer stage (American Joint Committee on Cancer—AJCC). The HR+/HER2– subtype was selected due to its high prevalence and limited sensitivity to neoadjuvant chemotherapy. Exploring how individual factors, such as gut microbiota composition, influence patients' mental health, cognitive function and QoL during and after treatment may reveal new dimensions of therapeutic response.

Exclusion criteria covered a range of factors such as: history of other malignancies (except for localized basal-cell carcinoma); prior treatment with chemotherapy, biologic treatment, endocrine therapy or radiotherapy for the treatment of the newly diagnosed breast cancer; history of any gastrointestinal tract surgery; any chronic inflammatory or autoimmune disease; major surgery within 28 days prior to registration for protocol study; signs or symptoms of infection within 2 weeks prior to registration for protocol study; Body Mass Index (BMI) > 30 Kg/m^2^; vegetarian or vegan diet; pregnant or lactating patients; recent (< 3 months prior) use of antibiotics, probiotics, prebiotics, symbiotics, laxatives, or Chinese herbal medicine; mental health disorder, dementia, and another clinical condition capable of influencing mental and cognitive status.

### 2.3 Sample, data collection and analysis

**Figure 1:**
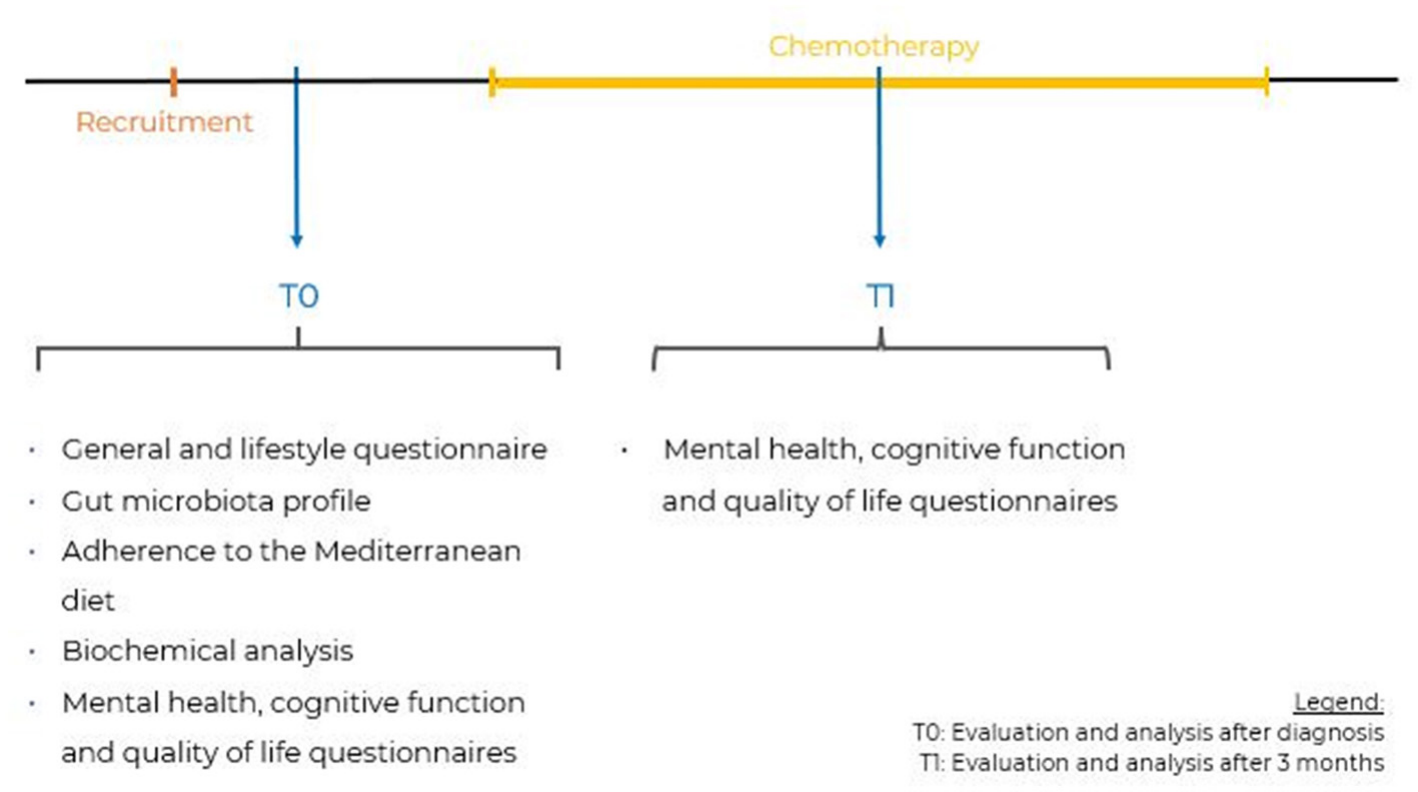
Representative diagram of the study timepoints.

Figure 1 represents a diagram of the study timepoints.

To assess the microbiota profile, including alpha-diversity (Shannon index), richness, and relative abundance of *phyla* and *genera*, participants provided one stool sample from up to 48 h beforehand, using the stool sampling kit provided by the research team. Stool samples were collected using EasySampler^®^ Stool Collection Kit (ALPCO) and maintained in RNAlater (Sigma). Samples were stored at −80 °C until analysis. The samples were collected following standardized protocols to ensure the preservation of microbial integrity, and stored under optimal conditions.

#### 2.3.1 16S rRNA gene amplification and sequencing

Genomic DNA was extracted and purified from stool samples using the NZY Tissue gDNA Isolation Kit (NZYTech) according to manufacturer instructions. The V3-V4 hypervariable regions of the 16S rRNA gene were amplified using the Ion 16S™ Metagenomics Kit (Thermo Fisher Scientific) with Environmental Master Mix v2.0. This master mix is optimized for complex environmental samples. The kit employs two primer set pools (V2-4-8 and V3-6,7-9) designed for comprehensive bacterial identification, aiming to achieve genus or species-level resolution. Library preparation was performed using the Ion Plus Fragment Library Kit (Thermo Fisher Scientific), and template preparation was conducted using the Ion Chef System (Thermo Fisher Scientific). Sequencing was performed on the Ion GeneStudio S5 Plus platform (Thermo Fisher Scientific), targeting 50,000 reads per sample.

#### 2.3.2 Sequence data processing and analysis

Raw sequencing data (BAM files) were converted to FASTQ format using bamToFastq. Quality filtering was performed using cutadapt with a minimum length threshold of 80 bp. Length trimming to a uniform 280 bp was subsequently performed using fastx_trimmer. The DADA2 pipeline (version 1.20) was implemented in R (v4.1.0) for sequence quality control, denoising, and chimera removal. The following DADA2 parameters were used: truncLen = c(240; 160), maxN = 0, maxEE = c(2,2), truncQ = 2, and trimLeft = 15. The trimLeft = 15 parameter was implemented to account for adapter or primer sequences specific to the Thermo Fisher sequencing platform. Chimeras were removed using removeBimeraDenovo in consensus mode. Amplicon Sequence Variants (ASVs) were generated, and ASVs with a minimum abundance of 10 reads across all samples were retained for downstream analysis.

Taxonomic classification was performed against the SILVA database (version 138.1) using the naive Bayesian classifier method implemented in the DADA2 package, with a minimum bootstrap confidence threshold of 80%. Data integration and community analyses were conducted using the Phyloseq package (v1.36.0) in R. Prior to diversity analyses, samples were rarefied to an even sampling depth of 50,000 reads per sample to account for variation in sequencing depth.

Venous blood samples were collected fasting as part of the clinical practice for breast cancer patients. Additionally, a certified laboratory determined vitamin D levels in serum by a standard chemiluminescence protocol.

Adherence to the Mediterranean diet was evaluated using the PREDIMED, completed by investigators or health care professionals. PREDIMED is a 14-item questionnaire in which each question is scored with 1 if meeting the defined criteria for this type of food pattern. A score of ≥ 10 is considered a good adherence to the Mediterranean diet (Afonso et al., 2014).

General and lifestyle information, such as age, weight, height, race, birth delivery mode, breastfeeding mode, menopausal state, sleep, and training description, were collected by investigators or health care professionals.

Participant's weight was obtained using the InBody S10 or InBody 770, and height was measured using a stadiometer. BMI was calculated.

The European Organization for Research and Treatment of Cancer Quality-of-Life Questionnaire Core-30 (EORTC QLQ-C30, version 3; validated for the Portuguese population (Pais-Ribeiro et al., 2008) was used to assess the patients' health-related QoL. The EORTC QLQ-C30 is a self-completed questionnaire with 30 items, 28 of which are scored on a 4-point Likert response scale: 1 = not at all, 2 = a little, 3 = quite a bit, and 4 = very much. The scoring procedure was based on the EORTC Scoring Manual (Fayers et al., 2001). The cut-off points for the global and functioning scales were determined to be scores below 33.3%, indicating problems, and scores above 66.7%, indicating good functioning. In contrast, the cut-off points for the symptom scales were reversed, with scores below 33.3% indicating good functioning and above 66.7% indicating problems (Imran et al., 2019; Ahn et al., 2007).

For the assessment of cognitive function, the Montreal Cognitive Assessment (MoCA, version 7.1; validated for the Portuguese population [Freitas et al., 2010]) was applied by a psychologist. This tool is a rapid screening instrument for mild cognitive dysfunction, which assesses different cognitive domains: attention and concentration, executive functions, memory, language, visuoconstructional skills, conceptual thinking, calculations, and orientation. The total possible score is 30 points, with scores of 26 or higher indicating normal cognitive function, 18 to 25 indicating mild cognitive impairment, 10 to 17 indicating moderate cognitive impairment, and less than 10 indicating severe cognitive impairment (Nasreddine, 2010).

Anxiety and depression were assessed through the Hospital Anxiety and Depression Scale (HADS; validated for the Portuguese population [Pais-Ribeiro et al., 2007]). This is a brief self-completed 14-item questionnaire, with seven items each for the anxiety and depression subscales. Responses are scored on a scale of 0 to 3, with total scale scores ranging from 0 to 21. Scores between 0 and 7 are within the normal range, scores between 8 and 10 are considered borderline abnormal, and scores between 11 and 21 are abnormal (Osborne et al., 2004; Stern, 2014).

### 2.4 Statistical methods

The descriptive statistics in this study are reported as numbers and percentages for categorical variables, mean and standard deviation (SD) for continuous variables, or median and interquartile range (IQR) in the case of skewed data.

The Shapiro-Wilk test was used to test for normality assumptions of the variable distributions. The Wilcoxon test was employed to compare the cognitive function, anxiety and depression, and QoL questionnaire scores at the two different timepoints. The Chi-square test and Fisher's Exact Test were appropriate when testing hypotheses about categorical variables, namely the evolution of questionnaire scores from baseline to the 3-month mark. Additionally, to explore the relationship between the questionnaire scores evolution overtime and the microbiota's alpha-diversity, richness, and relative abundance of *phyla* and *genera*, the one-way ANOVA test, Kruskall–Wallis test, Independent Samples *t*-test or Mann-Whitney test were conducted, according to the normality of variables. Differences were considered statistically significant when *p* < 0.05. Statistical analysis and graphs were performed using statistical SPSS Statistics version 28.

## 3 Results

### 3.1 Participant characteristics

A sample of 12 women with newly diagnosed HR+/HER2- breast cancer was recruited. One participant withdrew from the study as the cancer center of her treatments changed. The eleven women recruited had an average age of 51 ± 11 years, were all Caucasian, and 70% were overweight. Additionally, all participants were born by vaginal delivery, and 87.5% were breastfed. Regarding the patients' menopausal state, 75% were in pre-menopause, while the remaining 25% were in post-menopause. Most women (62.5%) slept between 6 and 8 h and did not exercise (Table 1).

**Table 1:** Participants' characterization.

**Characteristics**		**Result**	***n***
Age (years)		51.36 ± 11.04	11
Race	Caucasian	9 (100.0%)	9
	Black	0 (0.0%)	
	Indian	0 (0.0%)	
	Asian	0 (0.0%)	
	Mixed	0 (0.0%)	
	Another	0 (0.0%)	
BMI (kg/m^2^)	<18	0 (0.0%)	10
	[18; 25]	3 (30.0%)	
	[25; 30]	7 (70.0%)	
Smoking frequency (pack/year)	Never smoked	5 (55.6%)	9
	<10	2 (22.2%)	
	[10; 30]	1 (11.1%)	
	≥30	1 (11.1%)	
Alcohol consumption	Abstinent	2 (25.0%)	8
	Low-moderate (< 5 units weekly)	0 (0.0%)	
	Binge (occasional)	6 (75.0%)	
	Binge (frequent)	0 (0.0%)	
	Heavy	0 (0.0%)	
Training description	No exercise	5 (62.5%)	8
	Occasional (light exercise—once a week or once every 2 weeks)	2 (25.0%)	
	Regular (2 to 5 times per week)	1 (12.5%)	
	Daily	0 (0.0%)	
Sleep duration (hours)	<4h	0 (0.0%)	8
	[4; 6]	2 (25.0%)	
	[6; 8]	5 (62.5%)	
	≥8	1 (12.5%)	
Contraceptive agents use	<6 months	0 (0.0%)	8
	≥6 months	4 (50.0%)	
	Never	4 (50.0%)	
Menopausal state	Pre-menopause	6 (75.0%)	8
	Perimenopause	0 (0.0%)	
	Post-menopause	2 (25.0%)	
Mode of delivery	Cesarean section	0 (0.0%)	8
	Vaginal	8 (100.0%)	
	Do not know	0 (0.0%)	
Breastfeeding	Yes	7 (87.5%)	8
	No	1 (12.5%)	
	Do not know	0 (0.0%)	
Bowel habits change in the last 6 months	Yes	2 (25.0%)	8
	No	6 (75.0%)	
Antibiotics in the last 3 months	Yes	1 (14.3%)	7
	No	6 (85.7%)	
Chronic medication in the last 6 months	Yes	4 (50.0%)	8
	No	4 (50.0%)	
Medical conditions in the last 6 months	Yes	4 (50.0%)	8
	No	4 (50.0%)	
Score of the adherence to the Mediterranean diet	<10	10 (100.0%)	10
	≥10	0 (0.0%)	
Total cholesterol (mg/dL)	<190	1 (11.1%)	9
	≥190	8 (88.9%)	
Triglycerides (mg/dL)	<150	9 (100.0%)	9
	≥150	0 (0.0%)	
Vitamin D (ng/mL)	<10	0 (0.0%)	9
	[10; 30]	7 (77.8%)	
	[30; 100]	2 (22.2%)	

Participants' characterization at baseline. *n* = 7–11. Values are mean ± SD or *n* (%).

Analysis of the PREDIMED questionnaire results showed that all participants had a low adherence to the Mediterranean diet.

Biochemical analysis revealed several noteworthy findings. Among the patient cohort, 88.9% had total cholesterol levels above the recommended threshold of 190 mg/dL, indicating a high prevalence of hypercholesterolemia. However, all women showed adequate triglyceride levels below 150 mg/dL. Regarding vitamin D status, most women (77.8%) had insufficient serum levels ranging from 10 to 30 ng/mL, which is considered insufficiency.

### 3.2 Microbiota profile

**Figure 2:**
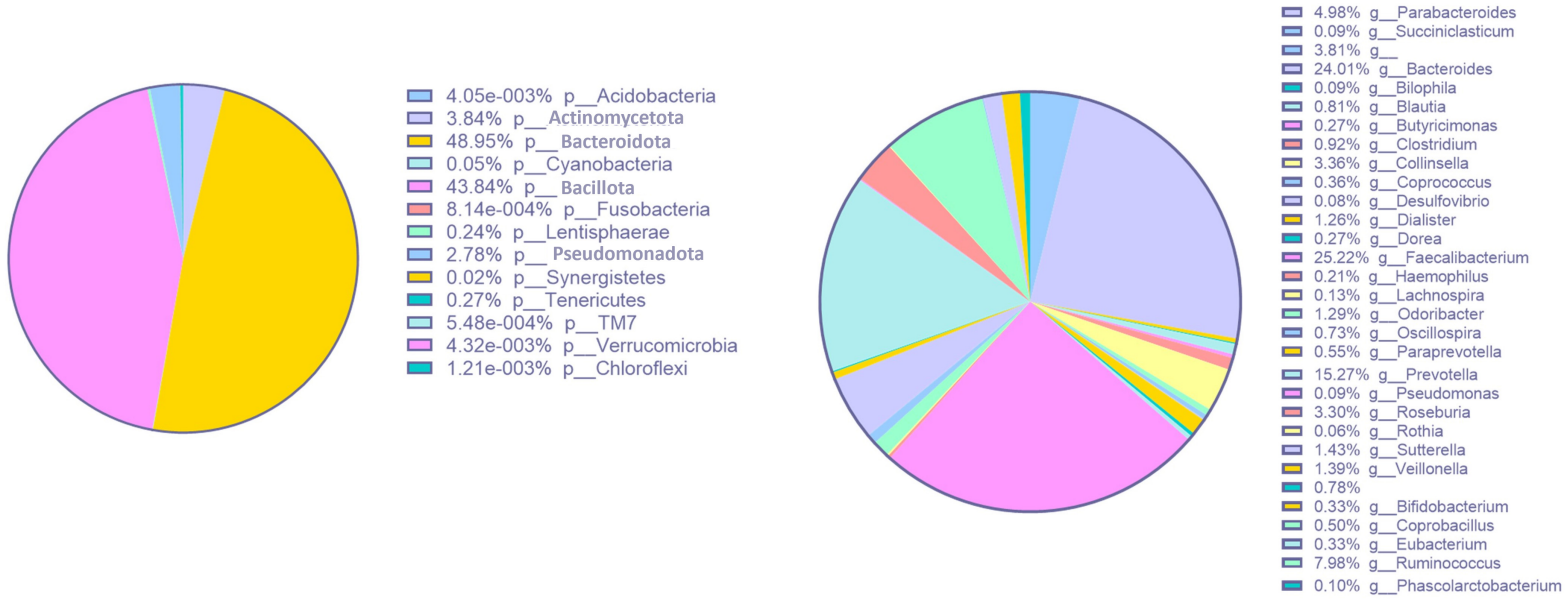
Representative diagrams of participants' relative abundance of *phyla* (p) and *genera* (g) at baseline.

**Figure 3:**
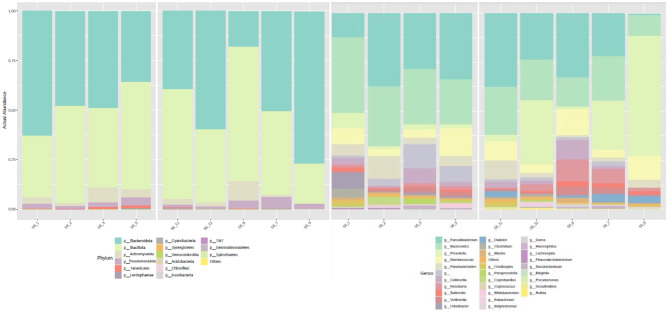
Representative diagrams of participants' relative abundance of *phyla* (p) and *genera* (g) at baseline, presented for each participant.

The microbiota profile analysis revealed a mean Shannon index of 2.52 ± 0.35 and a richness of 198 ± 24 (Table 2). The relative abundance of *phyla* is depicted in Figure 2, which displays a notable predominance of *Actinomycetota, Bacteroidota, Bacillota*, and *Pseudomonadota*. Regarding the relative abundance of *genera*, Figure 2 highlights *Bacteroides, Faecalibacterium, Prevotella*, and *Ruminococcus*. One participant presented a large relative abundance of the *genus Prevotella* compared to the others, as demonstrated in Figure 3.

**Table 2:** Characterization of participants' microbiota profile.

**Variable**	**Mean ±SD**	***n***
Diversity index (Shannon Index)	2.52 ± 0.35	9
Richness	198 ± 24	9

Characterization of participants' microbiota profile at baseline. n = 9. Values are mean ± standard deviation (SD).

### 3.3 Cognitive function

**Figure 4:**
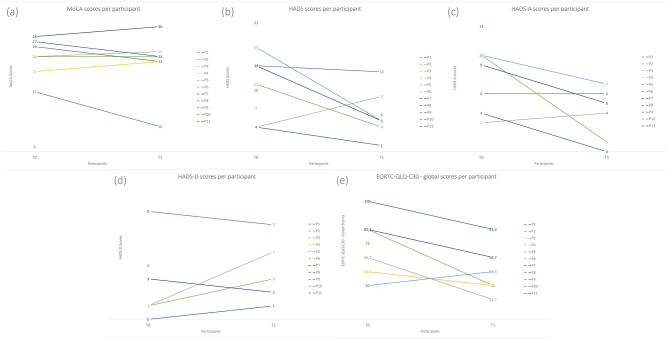
**(a–e)** Individual participant scores at baseline (T0) and follow-up (T1) for all questionnaires: MoCA, HADS (total score and subscales HADS-A and HADS-D), and EORTC QLQ-C30.

Regarding cognitive function, Supplementary Table 1 shows the results of cognitive function assessments conducted at baseline and the 3-month follow-up. The median score for all participants was 24 at both timepoints, indicating no significant changes in cognitive function over time. Figure 4a shows individual scores at each timepoint.

At baseline, 42.9% of participants scored above 25 on the MoCA questionnaire, while an equal percentage scored between 18 and 25, suggesting mild cognitive impairment. At the 3-month mark, the majority (71.4%) of patients scored between 18 and 25. Table 3 summarizes these results and highlights the decrease in the proportion of participants with normal cognitive function over time.

**Table 3:** Participants' distribution through Cognitive Function cut-off points, at baseline and 3 months after.

**Variable**		***n***	**T0**	**T1**	*p* ^*^
MoCA	< 10	7	0 (0.0%)	0 (0.0%)	0.078
	[10; 17]		1 (14.3%)	1 (14.3%)	
	[18; 25]		3 (42.9%)	5 (71.4%)	
	>25		3 (42.9%)	1 (14.3%)	

Participants' distribution through the Montreal cognitive assessment (MoCA) score cut-off points, at baseline (T0) and 3-months (T1). Higher scores indicate better cognitive function, scores bellow 10 indicate sever cognitive impairment. *n* = 7. Values are *n* (%).

^*^Chi-square test. *p*-value to assess differences in proportions across categories. Differences were considered significant if *p* < 0.05.

**Figure 5:**
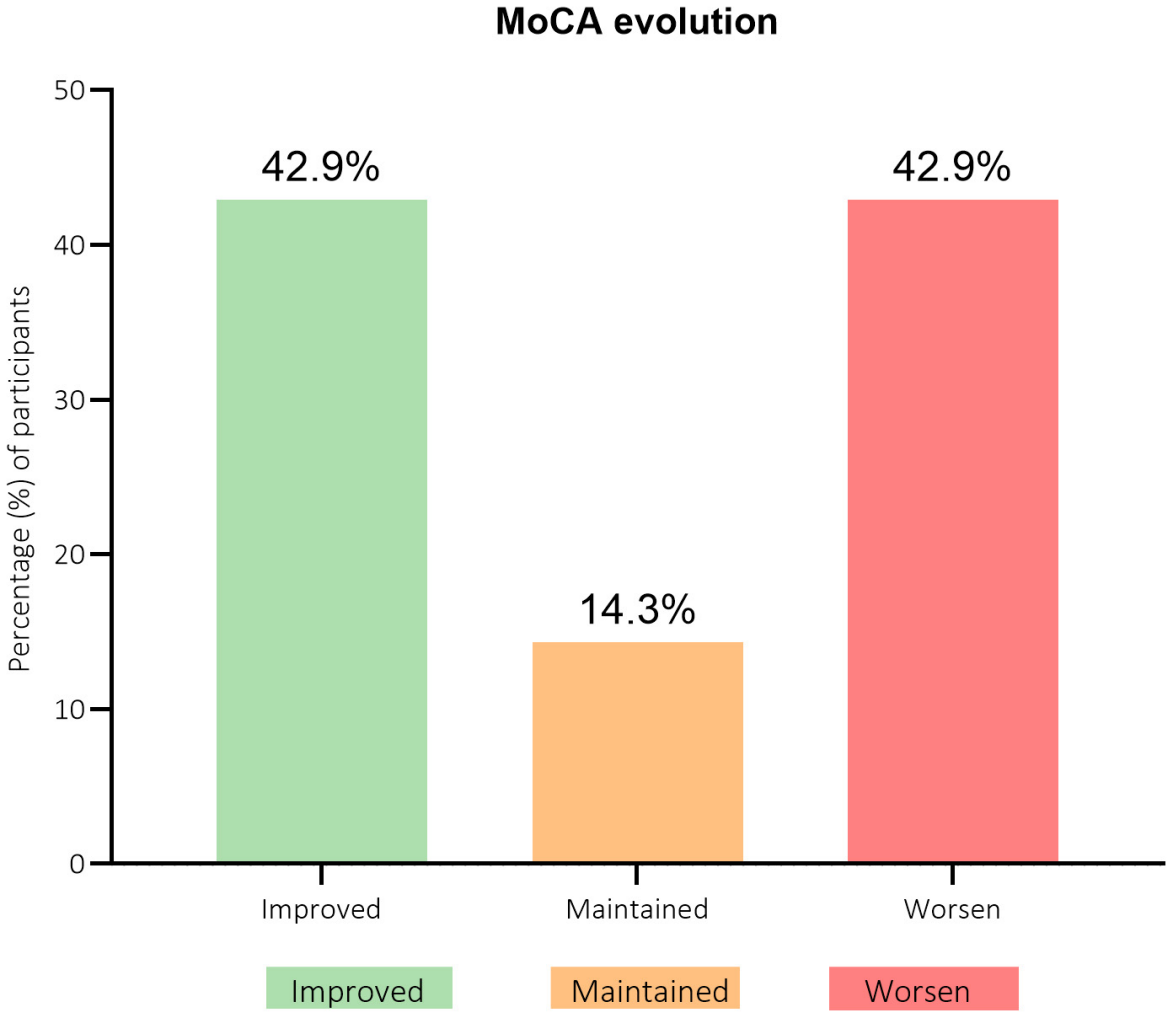
Participants' distribution through Montreal cognitive assessment (MoCA) questionnaire scores evolution from baseline (T0) to 3-months after (T1). *n* = 7.

However, further analysis of the data presented in Figure 5 revealed a complex pattern, with a comparable number of women showing improved or deteriorated cognitive function over time.

### 3.4 Anxiety and depression

For the HADS questionnaire, as demonstrated in Supplementary Table 2, the median scores seem to have decreased from T0 (13 [4–14]) to T1 (5 [4–9]) (*p* = 0.116). The median scores for anxiety seem to indicate a decrease from 8 [4–10] at T0 (suggesting possible anxiety) to 5 [1–6] at T1 (indicating improvement; *p* = 0.078). The depression subscale appears to show an increase from 2 [1–3] to 3 [2–5] (*p* = 0.518). Nevertheless, both indicate no depression. Figures 4b–d show individual scores at each timepoint, for the HADS questionnaire, and the anxiety and depression subscale, respectively.

The distribution of participants across the three HADS intervals is depicted in Table 4, which shows balanced results at baseline, with each interval accounting for 33.3% of the sample. However, the 3-month follow-up evaluation showed an increase in scores between 0 and 7, with 66.7% of participants falling within this range, meaning improvement. The remaining 33.3% of patients scored between 8 and 10, indicating borderline abnormality.

**Table 4:** Participants' distribution through Anxiety and Depression cut-off points, at baseline and 3 months after.

**Variable**		**n**	**T0**	**T1**	p^*^
HADS	[0; 7]	6	2 (33.3%)	4 (66.7%)	0.572
	[8; 10]		2 (33.3%)	2 (33.3%)	
	[11; 21		2 (33.3%)	0 (0.0%)	
HADS—anxiety	[0; 7]	6	3 (50.0%)	6 (100.0%)	-
	[8; 10]		3 (50.0%)	0 (0.0%)	
	[11; 21]		0 (0.0%)	0 (0.0%)	
HADS—depression	[0; 7]	6	5 (83.8%)	6 (100.0%)	-
	[8; 10]		1 (16.7%)	0 (0.0%)	
	[11; 21]		0 (0.0%)	0 (0.0%)	

Participants' distribution through the hospital anxiety and depression scales (HADS) score cut-off points, at baseline (T0) and 3-months (T1). Lower scores indicate normality, scores between 11 and 21 indicate abnormal levels of anxiety or depression. *n* = 6. Values are n (%).

^*^Chi-square test. *p*-value to assess differences in proportions across categories. Differences were considered significant if *p* < 0.05.

The anxiety subscale demonstrated that, at baseline, the participants were divided into scores between 0 and 7 (50.0%) and 8 and 10 (50.0%), while at T1, all women scored between 0 and 7. Regarding the depression scores, most patients scored between 0 and 7, both at baseline (83.8%) and the 3-month mark (100.0%).

**Figure 6:**
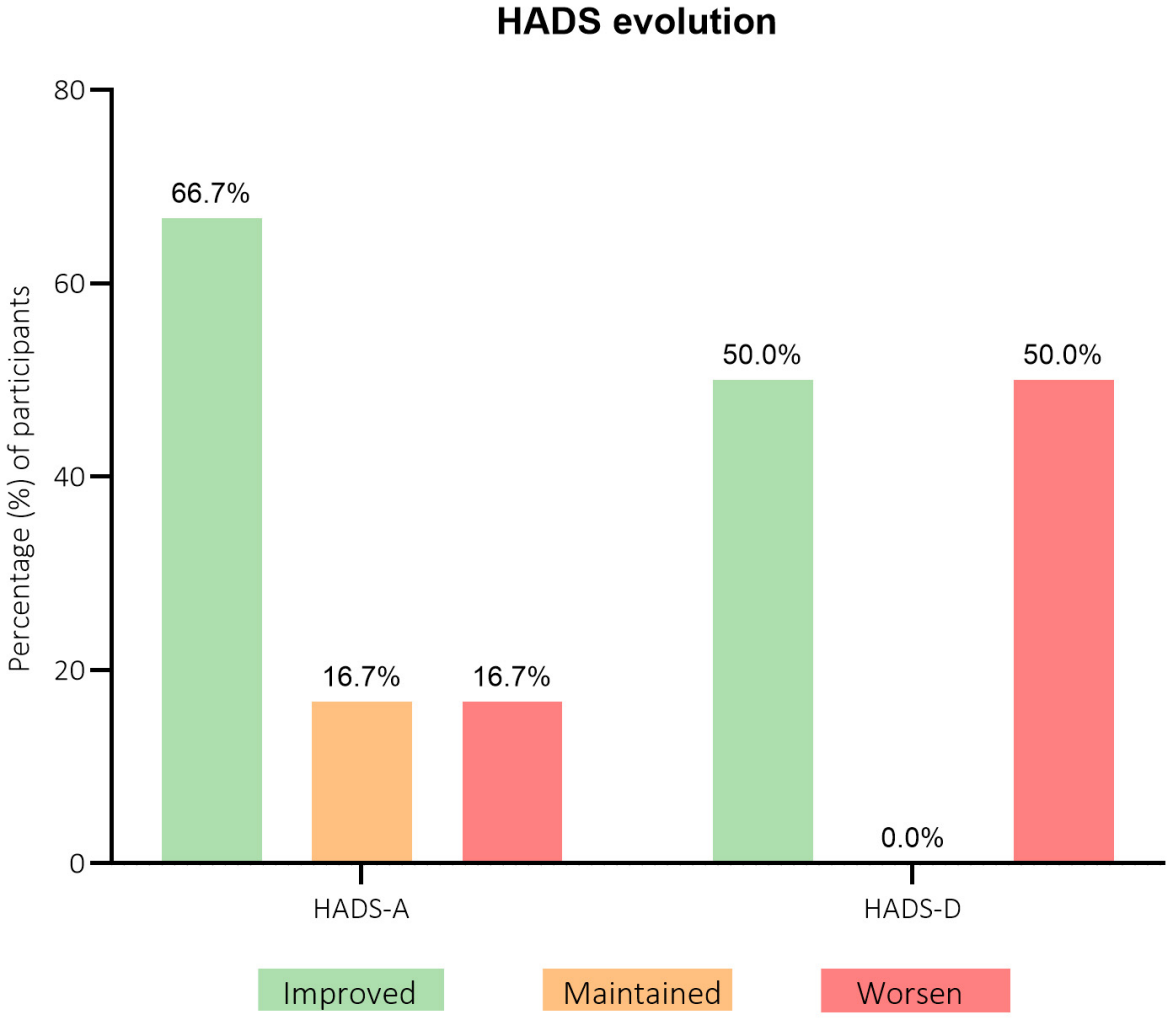
Participants' distribution through hospital anxiety and depression scales (HADS) questionnaire scores evolution from baseline (T0) to 3-months after (T1). *n* = 6.

Figure 6 demonstrates a tendency for improvement (83.3%) in HADS scores. As for the anxiety and depression subscales, the results show that 66.7% of patients reported an improvement in the anxiety subscale. For the depression subscale, 50.0% reported an improvement, and the same percentage of individuals worsened their pre-treatment scores, with no participant maintaining their depression scores.

### 3.5 Quality-of-life

Results from the global scale of the EORTC QLQ-C30 questionnaire show a significant decrease from baseline (67 [58–83]) to the 3-month mark (50 [42–67]). Figure 4e shows individual scores at each timepoint.

Regarding the functional scales, median scores at T0 were highest for physical (93 [87–100]) and role (which reflects a patient's ability to perform daily activities such as work and hobbies) (100 [83–100]). By T1, the highest median scores were observed for emotional (92 [83–92]) and cognitive (89 [83–94]) scales, while social function scores remained unchanged. For the symptom scales, higher scores were generally observed at T1 compared to T0, particularly for fatigue (33 [22–33]), nausea (8 [0–17]), diarrhea (33 [0–33]), and financial difficulties (33 [0–33]). The remaining symptom scales showed no change in median scores between assessments. Statistically significant changes were observed only for fatigue and diarrhea (Supplementary Table 3).

Table 5 shows the distribution of participants through the QoL scores' cut-off points. At baseline, most participants (85.7%) scored above 66.7 for global health status, indicating good functioning. However, at the 3-month follow-up evaluation, the proportion of participants with scores above 66.7 decreased sharply to 28.6%, while those scoring between 33.3 and 66.7 increased to 71.4%. The results seem to reveal an overall reduction in the global scale scores (85.7%), except for one woman who showed improvement.

**Table 5:** Participants' distribution through quality-of-life cut-off points, at baseline and 3 months after.

**Variable**		***n***	**T0**	**T1**	*p* ^*^
EORTC-QLQ-C30—global	< 33.3	7	0 (0.0%)	0 (0.0%)	1.000^a^
	[33.3; 66.7]		1 (14.3%)	5 (71.4%)	
	≥66.7		6 (85.7%)	2 (28.6%)	
**EORTC-QLQ-C30—functioning scales**					
Physical	< 33.3	7	0 (0.0%)	0 (0.0%)	-
	[33.3; 66.7]		0 (0.0%)	0 (0.0%)	
	≥66.7		7 (100.0%)	7 (100.0%)	
Role	< 33.3	6	0 (0.0%)	0 (0.0%)	-
	[33.3; 66.7]		0 (0.0%)	0 (0.0%)	
	≥66.7		6 (100.0%)	6 (100.0%)	
Emotional	< 33.3	7	0 (0.0%)	0 (0.0%)	-
	[33.3; 66.7]		0 (0.0%)	0 (0.0%)	
	≥66.7		7 (100.0%)	7 (100.0%)	
Cognitive	< 33.3	7	0 (0.0%)	0 (0.0%)	-
	[33.3; 66.7]		0 (0.0%)	0 (0.0%)	
	≥66.7		7 (100.0%)	7 (100.0%)	
Social	< 33.3	7	0 (0.0%)	0 (0.0%)	-
	[33.3; 66.7]		0 (0.0%)	1 (14.3%)	
	≥66.7		7 (100.0%)	6 (85.7%)	
**EORTC-QLQ-C30—symptom scales**					
Fatigue	< 33.3	7	6 (85.7%)	3 (42.9%)	1.000^a^
	[33.3; 66.7]		1 (14.3%)	4 (57.1%)	
	≥66.7		0 (0.0%)	0 (0.0%)	
Nausea	< 33.3	6	6 (100.0%)	6 (100.0%)	-
	[33.3; 66.7]		0 (0.0%)	0 (0.0%)	
	≥66.7		0 (0.0%)	0 (0.0%)	
Pain	< 33.3	7	6 (85.7%)	7 (100.0%)	-
	[33.3; 66.7]		1 (14.3%)	0 (0.0%)	
	≥66.7		0 (0.0%)	0 (0.0%)	
Dyspnea	< 33.3	7	7 (100.0%)	7 (100.0%)	-
	[33.3; 66.7]		0 (0.0%)	0 (0.0%)	
	≥66.7		0 (0.0%)	0 (0.0%)	
Insomnia	< 33.3	7	3 (42.9%)	2 (28.6%)	0.323^b^
	[33.3; 66.7]		3 (42.9%)	4 (57.1%)	
	≥66.7		1 (14.3%)	1 (14.3%)	
Appetite	< 33.3	7	6 (85.7%)	5 (71.4%)	0.286^a^
	[33.3; 66.7]		1 (14.3%)	2 (28.6%)	
	≥66.7		0 (0.0%)	0 (0.0%)	
Constipation	< 33.3	7	4 (57.1%)	6 (85.7%)	1.000^a^
	[33.3; 66.7]		3 (42.9%)	1 (14.3%)	
	≥66.7		0 (0.0%)	0 (0.0%)	
Diarrhea	< 33.3	7	7 (100.0%)	3 (42.9%)	-
	[33.3; 66.7]		0 (0.0%)	4 (57.1%)	
	≥66.7		0 (0.0%)	0 (0.0%)	
Financial difficulties	< 33.3	7	6 (85.7%)	3 (42.9%)	1.000^a^
	[33.3; 66.7]		1 (14.3%)	4 (57.1%)	
	≥66.7		0 (0.0%)	0 (0.0%)	

Participants' distribution through the European Organization for Research and Treatment of Cancer Quality-of-Life Questionnaire Core-30 (EORTC QLQ-C30) score cut-off points, at baseline (T0) and 3-months (T1). For the global and functional scales, scores below 33.3% indicate problems, and scores above 66.7% indicate good functioning. For the symptom scales scores above 66.7% indicate problems, and scores below 33.3% indicate good functioning. *n* = 6–7. Values are n (%).

^*^Fisher's Exact test^a^ or Chi-square test^b^. *p*-value to assess differences in proportions across categories. Differences were considered significant if *p* < 0.05.

The functional scales exhibited consistent scores across both timepoints, with the vast majority of participants scoring above 66.7%. In contrast, the symptom scales showed different patterns. At baseline, most women scored below 33.3% at T0, with the exception of insomnia, which had 42.9% of participants scoring both below 33.3% and between 33.3% and 66.7%. However, at the 3-month follow-up, several symptom scales showed increased percentages for scores between 33.3% and 66.7%, including fatigue, insomnia, diarrhea, and financial difficulties. In contrast, the majority of scores for nausea, pain, dyspnea, appetite, and constipation remained below 33.3%.

**Figure 7:**
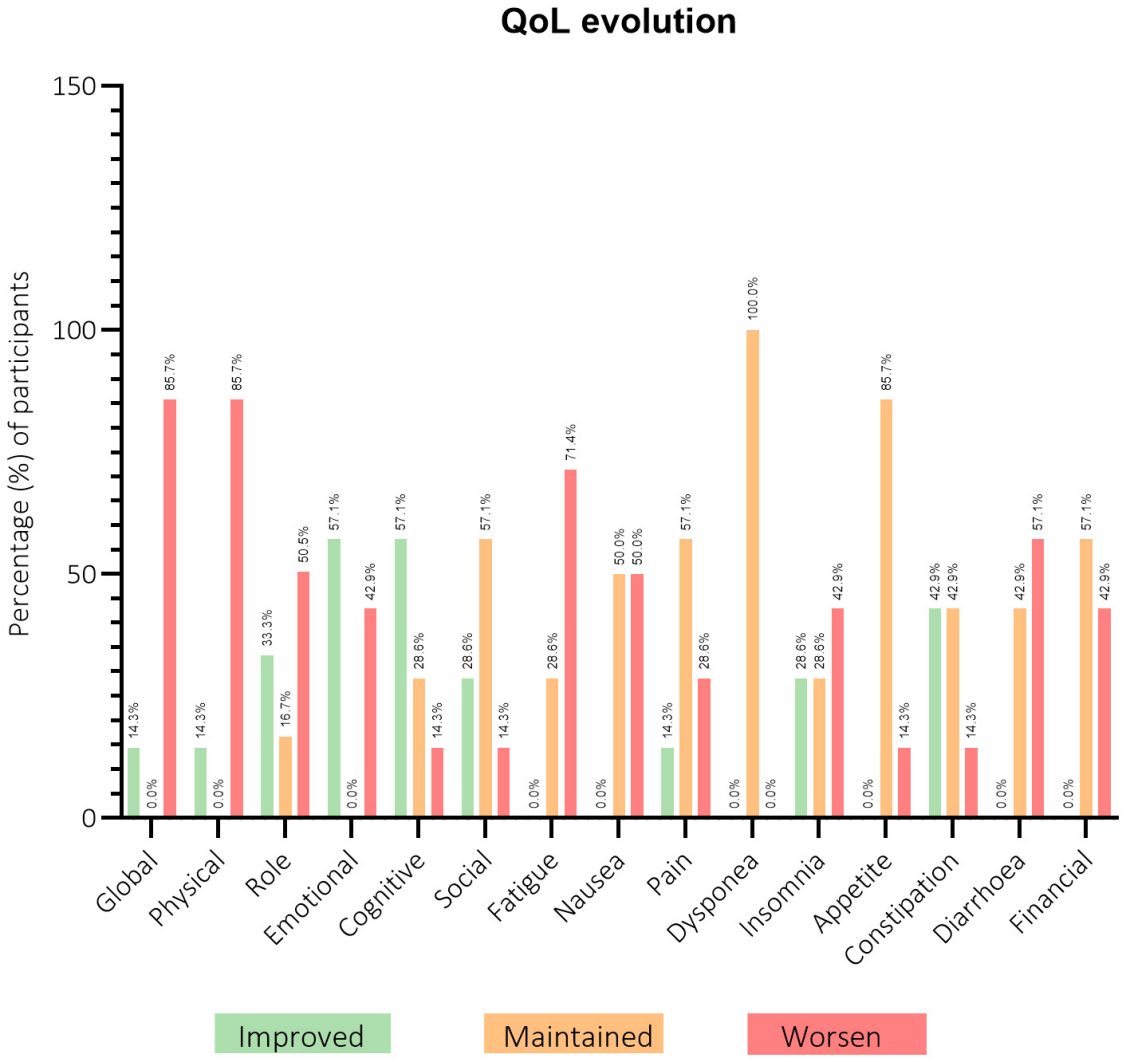
Participants' distribution through European Organization for Research and Treatment of Cancer Quality-of-Life Questionnaire Core-30 (EORTC QLQ-C30) questionnaire scores evolution from baseline (T0) to 3-months after (T1). *n* = 6–7.

Figure 7 provides a comprehensive overview of the changes in the various scales from baseline to the 3-month mark (T1). Regarding the functional scales, most women on the physical scale presented worse scores, while the emotional and cognitive scales had improved scores. In the role scale, half of the women experienced worsening scores, while 33.3% showed improvement. The social scale had the majority of participants maintaining their scores. On the symptom scales, there were mixed results, with a tendency for participants to maintain their scores, namely in areas such as pain, dyspnea, appetite, and financial difficulties. The only scale with the majority of women having worsening scores was fatigue.

### 3.6 Correlation between gut microbiota profile and cognitive function, mental health and quality-of-life

Our study also aimed to examine if the patients' cognitive function, anxiety and depression, and QoL changes after 3 months of treatment were associated with their initial gut microbiota profile, and the results are presented in Supplementary Table 4. Given the small sample size, statistical testing was not performed. Therefore, from this point onward, the results are interpreted primarily in terms of observed tendencies and descriptive patterns.

There was no relationship between changes in cognitive function on the MOCA or psychological symptoms on the HADS and alpha diversity measures. The results indicate that women with poorer cognitive function over time tended to have lower Shannon index and richness. On the other hand, the Shannon index of the microbiota was similar for the HADS score evolution but tended to be higher in people with worsening symptoms. However, the opposite seems to be true for richness.

Regarding the anxiety subscale, the data indicates, with no statistical significance, that Shannon index was higher for women who maintained their scores over time, with the next highest Shannon index observed in those who experienced a decline in scores. In contrast, richness tended to be higher in women who improved their scores over time, with the second-highest richness observed in those whose scores worsened. The results of the depression subscale analysis appear to indicate that Shannon index was higher in patients who experienced an improvement in scores from baseline to the 3-month mark. However, richness analysis showed the highest value in women whose scores worsened over time. Notably, none of the participants maintained their scores between the two timepoints.

The study also explored the relationship between QoL and microbiota profile. Women with improved global QoL scores appear to have lower Shannon index but higher richness. For the functioning scales, Shannon index was higher in patients who had improved physical and social scales, but also in women who had worsened in the role and emotional scales, and who maintained the score for the cognitive scale. In terms of richness, patients with worsening symptoms on the physical scale had higher richness. Additionally, the remaining scales appear to demonstrate higher richness in women who improved in these parameters over time.

In contrast, for the symptom scales, most seem to show higher Shannon index when scores were maintained, except for appetite and financial difficulties, in which the highest Shannon index was found in worsened scores. Furthermore, for constipation, a higher Shannon index was found in people who had improved symptoms and lower Shannon index in women with worsening symptoms. Women who maintained their scores over time for fatigue, nausea, dyspnea, constipation, and diarrhea seem to present higher richness. Apart from that, except for the pain scale, which was higher for those who improved from baseline to the 3-month mark, the remaining scales had higher richness for those who worsened.

The correlation between participants' microbiota profile and the questionnaires scores at baseline and 3 months after, was demonstrated in Table 6.

**Table 6:** Correlation between participants' microbiota profile and the Cognitive Function, Mental Health and QoL scores at baseline and 3 months after.

**Questionnaires**	***n***	**Alpha-diversity**	*p* ^*^	**Richness**	*p* ^*^
MoCA T0	8	−0.133	0.754	0.446	0.268
MoCA T1	8	−0.233	0.578	0.147	0.728
HADS T0	7	0.418	0.350	0.491	0.263
HADS T1	7	0.505	0.248	−0.414	0.355
HADS—anxiety T0	7	0.216	0.641	0.829	**0.021**
HADS—anxiety T1	8	0.277	0.506	−0.193	0.647
HADS—depression T0	7	0.753	0.051	0.128	0.784
HADS—depression T1	7	0.630	0.129	−0.445	0.317
EORTC-QLQ-C30—global T0	8	−0.061	0.887	−0.206	0.624
EORTC-QLQ-C30—global T1	8	−0.352	0.393	0.509	0.197
**EORTC-QLQ-C30—functioning scales**					
Physical T0	8	−0.284	0.495	0.531	0.175
Physical T1	8	0.443	0.271	0.039	0.927
Role T0	7	−0.040	0.932	−0.578	0.174
Role T1	8	−0.432	0.285	−0.247	0.555
Emotional T0	8	0.203	0.630	−0.723	**0.043**
Emotional T1	8	−0.526	0.181	−0.300	0.470
Cognitive T0	8	0.135	0.750	−0.602	0.115
Cognitive T1	8	−0.246	0.558	−0.282	0.498
Social T0	8	−0.082	0.846	−0.619	0.102
Social T1	8	0.247	0.555	0.412	0.310
**EORTC-QLQ-C30—symptom scales**					
Fatigue T0	8	0.247	0.555	0.964	**< 0.001**
Fatigue T1	8	0.025	0.953	0.025	0.953
Nausea T0	7	-	-	-	-
Nausea T1	8	−0.104	0.806	−0.183	0.665
Pain T0	8	0.031	0.942	−0.312	0.452
Pain T1	8	0.209	0.620	0.274	0.512
Dyspnea T0	8	-	-	-	-
Dyspnea T1	8	-	-	-	-
Insomnia T0	8	0.202	0.632	−0.113	0.789
Insomnia T1	8	−0.039	0.927	0.117	0.782
Appetite T0	8	0.082	0.846	0.412	0.310
Appetite T1	8	0.126	0.766	0.252	0.547
Constipation T0	8	0.507	0.200	−0.507	0.200
Constipation T1	8	−0.378	0.356	0.252	0.547
Diarrhea T0	8	0.247	0.555	0.577	0.134
Diarrhea T1	8	0.169	0.689	0.056	0.895
Financial difficulties T0	8	0.126	0.766	0.378	0.356
Financial difficulties T1	8	−0.169	0.689	0.282	0.499

Correlation between participants' Shannon Index and richness and the Montreal cognitive assessment (MoCA), hospital anxiety and depression scales (HADS) and European Organization for Research and Treatment of Cancer Quality of Life Questionnaire Core-30 (EORTC QLQ-C30) scores at baseline (T0) and 3-months after (T1). *n* = 7–8.

^*^Spearman correlation coefficient. Differences were considered significant if *p* < 0.05. Bold data indicate statistically significant *p*-values.

**Figure 8:**
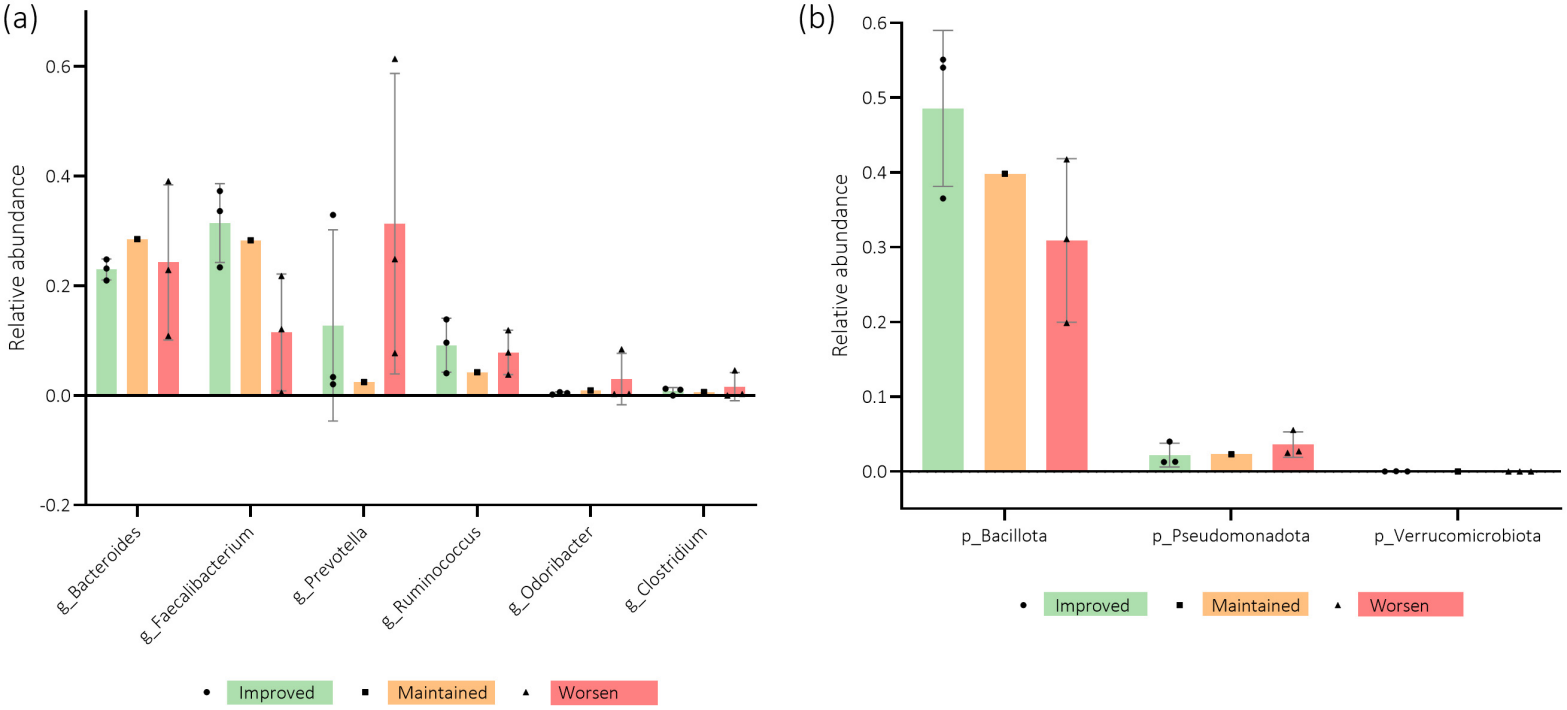
**(a, b)** Participants' microbiota relative abundance of *genera* (g) and *phyla* (p) for the Montreal Cognitive Assessment (MoCA) questionnaire score evolution from baseline (T0) to 3-months after (T1). *n* = 7. Error bars indicate standard deviation (SD).

The association of the relative abundance of the microbiota, namely *phyla* and *genera*, and MoCA score evolution from baseline to the 3-month mark is depicted in Figures 8a, b. The women who improved their cognitive function appear to have increased relative abundance of the *phyla Bacillota, Verrucomicrobia*, and *genera Faecalibacterium, Ruminococcus*, and *Clostridium*. *Bacteroides* and *Odoribacter* tended to be more present in participants who maintained their scores, while *Prevotella* and *Pseudomonadota* were the ones who showed the worst cognitive function over time.

**Figure 9:**
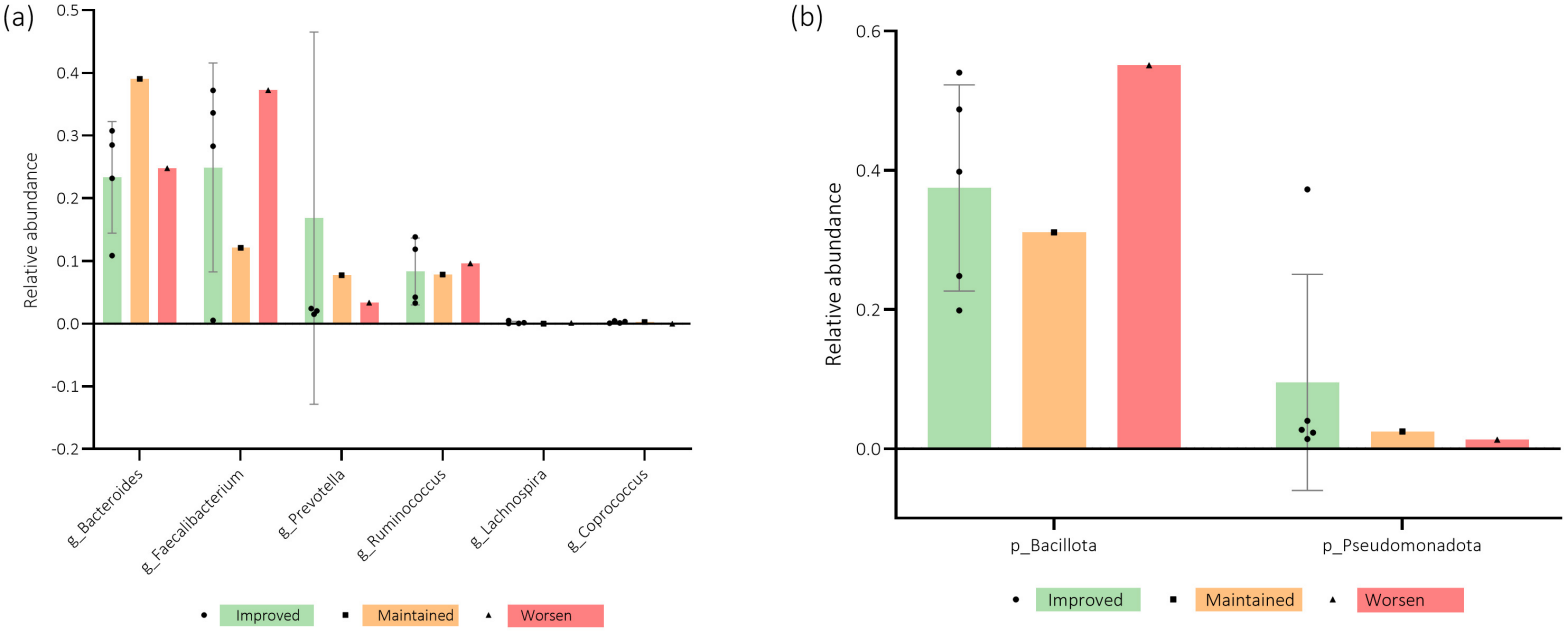
**(a, b)** Participants' microbiota relative abundance of *genera* (g) and *phyla* (*p*) for the Hospital Anxiety and Depression Scale Anxiety subscale (HADS-A) questionnaire score evolution from baseline (T0) to 3-months after (T1). *n* = 6. Error bars indicate standard deviation (SD).

Upon analyzing Figures 9a, b, it is possible to see that an increased relative abundance of *Pseudomonadota* seems to be present in participants with better scores for the anxiety subscale of the HADS questionnaire. However, the *genera Bacteroides, Prevotella*, and *Coprococcus* appear to be more present in women who maintained their scores. The patients who worsened over time tended to present increased *Bacillota, Faecalibacterium, Ruminococcus*, and *Lachnospira* levels.

**Figure 10:**
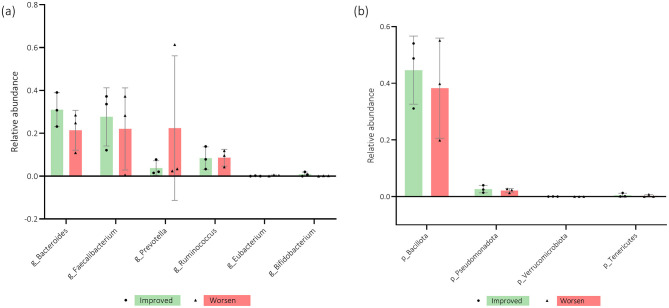
**(a, b)** Participants' microbiota relative abundance of *genera* (g) and *phyla* (*p*) for the Hospital Anxiety and Depression Scale Depression subscale (HADS-D) questionnaire score evolution from baseline (T0) to 3-months after (T1). *n* = 6. Error bars indicate standard deviation (SD).

Regarding the association of relative abundance with the HADS depression subscale, Figures 10a, b demonstrate that increased levels of *Bacteroides, Bacillota, Pseudomonadota, Verrucomicrobia, Tenericutes*, and *Bifidobacterium* tended to be found in women who improved over time. However, *Prevotella, Ruminococcus*, and *Eubacterium* abundance appears to be higher in participants whose depression scores worsened from T0 to T1.

**Figure 11:**
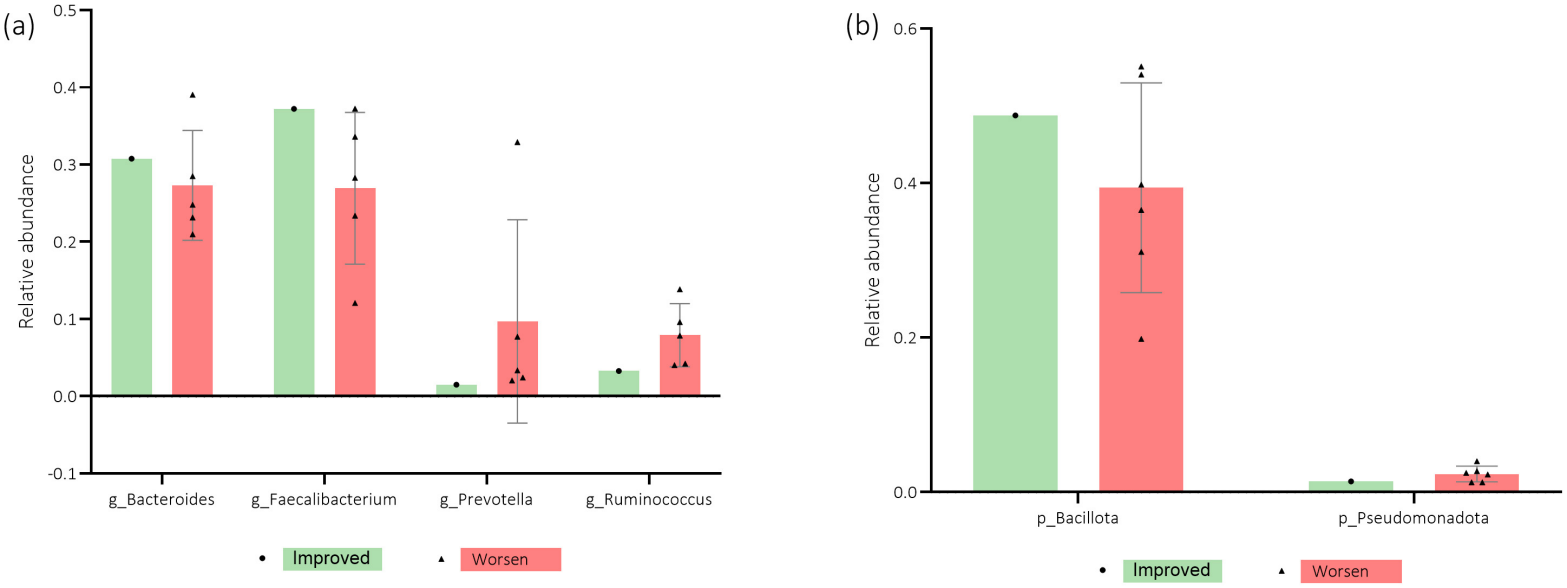
**(a, b)** Participants' microbiota relative abundance of *genera* (g) and *phyla* (p) for the European Organization for Research and Treatment of Cancer Quality-of-Life Questionnaire Core-30 (EORTC QLQ-C30) questionnaire score evolution from baseline (T0) to 3-months after (T1). *n* = 6–7. Error bars indicate standard deviation (SD).

Figures 11a, b highlight EORTC QLQ-C30 Global score evolution associations with microbiota's relative abundance. Patients whose QoL improved over time tended to have higher levels of *Bacteroides, Bacillota*, and *Faecalibacterium*, while those whose QoL worsened from T0 to T1 appear to have a higher relative abundance of *Prevotella, Ruminococcus*, and the *phylum Pseudomonadota*.

## 4 Discussion

The existing literature demonstrates that cancer and anti-cancer treatments have several adverse effects, both physical and psychological—including anxiety, depression, altered cognitive function, and loss of QoL—and that the microbiota may play an important role in their emergence. This is an essential topic since these adverse effects, often neglected, significantly impact patients' daily functioning, adherence to treatment, treatment outcomes and costs, and survival (Subramaniam et al., 2020; Rodríguez Martín et al., 2020; Pitman et al., 2018; Smith, 2015; Wefel et al., 2011). Given the growing number of breast cancer survivors, a better understanding of the patients' response to treatment could enable a more personalized intervention, with the maintenance and restoration of a healthy microbiota before, during, and after treatment as an important step to improve clinical outcomes and the emergence of adverse effects (Sampsell et al., 2020; Alpuim Costa et al., 2021; Ciernikova et al., 2021; Bajic et al., 2018).

Our study revealed that breast cancer patients could potentially experience changes in cognitive function, anxiety, depression, and QoL and that there may be a correlation between these factors and the patients' gut microbiota profile at diagnosis. Although no statistically significant differences were found for most outcomes, this is an exploratory study with a small sample size, which limits the ability to detect significant differences, even if they exist. Nonetheless, differences were observed for some parameters.

Most participants showed normal or mildly impaired cognitive function, consistent with prior studies (Pereira et al., 2015). The literature also shows that, at baseline, cognitive dysfunction was present in the minority of participants, with no significant changes over time (Iconomou et al., 2004). Chemotherapy may mildly but significantly affect cognitive function in breast cancer patients (Pereira et al., 2015; Ramalho et al., 2017; Falleti et al., 2005; Stewart et al., 2006, 2008; Hermelink et al., 2007; Lopes-Conceição et al., 2021). Women treated with doxorubicin and cyclophosphamide (with or without taxanes)—which are the treatment regimens used in our sample—show a higher risk of impairment (Ramalho et al., 2017), though timing differences from our study may explain inconsistencies, as cognitive decline may not be evident after only three months (Stewart et al., 2008; Hermelink et al., 2007; Bilenduke et al., 2022).

Evidence shows both an increase or decrease in cognitive function over time following chemotherapy (Hermelink et al., 2007; Fontes et al., 2016; Jenkins et al., 2006; Dijkshoorn et al., 2021), similarly to our results. To note that practice effects were not considered (McCabe et al., 2011), and that early cognitive impairment may reflect diagnosis-related distress, which tends to lessen within a year (Araújo et al., 2021). On the same note, advancements reducing chemotherapy toxicity may explain the absence of marked cognitive deficits, though cumulative effects could appear later. Evidence shows that 20% of women revealed consistently low or decreased MoCA scores over time (Araújo et al., 2021).

Our findings revealed that individuals with deteriorating cognitive function tended to have lower Shannon Index and richness. Our results seem to align with the limited research on this topic; however, some are in different populations. Studies in breast cancer survivors and healthy individuals have observed correlations between increased Shannon index and higher cognitive function (Deleemans et al., 2022; Tooley, 2020). On the same note, recent findings suggest a link between decreased microbial diversity and severe fear of cancer recurrence in breast cancer survivors, which involves similar brain regions to cognition (Okubo et al., 2020). Evidence has also associated lower richness with cognitive dysfunction. Patients undergoing frequent chemotherapy over extended periods may experience incomplete microbiota recovery, which has been associated with increased systemic inflammation. This heightened inflammatory state, compounded by the mental strain of treatment, could contribute to an elevated risk of cognitive impairment (Bilenduke et al., 2022). Evidence on microbiome richness differences between breast cancer and healthy controls is inconsistent; however, significant differences in overall cognitive functioning have been reported between these groups (Bilenduke et al., 2022).

Concerning the relative abundance of *genera* and *phyla*, we found that women who improved their cognitive function appear to have increased levels of *Bacillota, Verrucomicrobia, Faecalibacterium, Ruminococcus* and *Clostridium*. In contrast, those who worsen had higher levels of *Prevotella* and *Pseudomonadota*. Even though direct comparisons are limited to the scarce literature found, a study revealed that breast cancer patients, already subjected to chemotherapy, presented worst cognitive function than healthy controls, as well as lower levels of *Akkermansia* (*phylum Verrucomicrobia*) and increased *Clostridium*. Our results align with the possible association with *Verrucomicrobia* but not for *Clostridium*. Additionally, lower *Odoribacter* and higher *Clostridium* were associated with higher levels of cognitive impairment, findings that are not in line with ours (Bilenduke et al., 2022). In breast cancer survivors, the fear of cancer recurrence was higher in patients with a lower relative abundance of *Bacillota, Lachnospiraceae* and *Ruminococcus* and a higher relative abundance of *Bacteroides* (Okubo et al., 2020). Our results seem to agree with some of this evidence. Regardless, differences in study protocols hinder a direct comparison with our study. This evidence pertains to patients already undergoing treatment or survivors. Since the present study aimed to assess the impact of the microbiota profile at diagnosis on the emergence of cognitive dysfunction and psychological problems during treatment, we did not see the possible impact of the disease, chemotherapy and other conditions, on microbiota and cognitive function.

Evidence shows that breast cancer patients present higher levels of depression and anxiety compared to the general population, which is especially relevant for the ones going through chemotherapy (Bilenduke et al., 2022). While anxiety is considered the most common psychiatric disorder in breast cancer patients, and there is a large prevalence of depression, our results did not corroborate these findings (Ramalho et al., 2017; Maass et al., 2015). Some studies also report no significant differences from healthy controls (Maass et al., 2015; Lopes et al., 2022a). Regarding the evolution of anxiety levels, research suggests that its prevalence gradually decreases overtime, which our results corroborate (Iconomou et al., 2004; Ramalho et al., 2017; Lopes-Conceição et al., 2021; Araújo et al., 2021; Lopes et al., 2022a). However, other research show stable or worsening levels during follow-up (Maass et al., 2015; Lopes et al., 2022b; Crane et al., 2019).

Findings on anxiety and depression during chemotherapy are inconsistent. High baseline levels, apart from specific neuropsychiatric effects, may be related to the patient's reaction to the diagnosis. Later improvements may result from greater acceptance and resilience. However, evidence also shows its presence in a later stage, with the emergence of side effects associated with chemotherapy and, afterwards, with the transition to surveillance and the possibility of disease recurrence. At our 3-month assessment, this could not have happened yet, with still a chance for deterioration of the patients' mental health in the following months. Thereby, it is essential to signal the patients with higher levels of anxiety and depression at diagnosis, perform periodic follow-up assessments, and intervene (Pitman et al., 2018; Lopes-Conceição et al., 2021; Lopes et al., 2022b; Crane et al., 2019; Stark and House, 2000; Zhang et al., 2018).

Our findings suggest a possible relationship between symptoms of anxiety and depression and the microbiota profile. Specifically for anxiety, Shannon index was lower, and richness was higher when women improved their scores. The mismatch between alpha-diversity and richness may reflect reduced bacterial abundance with preserved species representation, though some dominant species could negatively affect health. On the contrary, women with improved scores for depression presented higher Shannon index and lower richness. The limited evidence suggests that cancer patients with anxiety and depression present reduced Shannon index compared with patients without these symptoms (Bilenduke et al., 2022; Zhu et al., 2021). Likewise, higher Shannon index and richness tended to be associated with less depressive symptoms (Maitiniyazi et al., 2022; Deleemans et al., 2022). However, studies in other populations have reported inconsistent results (Simpson et al., 2021). Evidence has demonstrated that patients with major depressive disorder tend to have equal or reduced Shannon index compared to the general population (Simpson et al., 2021; Sanada et al., 2020; Knuesel and Mohajeri, 2021; Nikolova et al., 2021). Lower richness in breast cancer patients has been linked to greater depressive symptoms, possibly due to incomplete microbiome recovery and increased inflammation, compounded by treatment-related stress (Simpson et al., 2021; Bilenduke et al., 2022; Nikolova et al., 2021). Ultimately, the literature points to a healthier microbiota, with higher alpha-diversity, being protective against the emergence of anxiety and depression. Inconsistencies in our anxiety subscale results may be attributed to the positive influence of other factors. Additionally, higher richness in this context suggests that certain bacteria maintain their representativeness, potentially positively influencing anxiety.

Considering the relative abundance of microbiota, a study assessing breast cancer patients undergoing chemotherapy and healthy controls found higher depressive symptoms in breast cancer patients, who also presented a lower relative abundance of *Akkermansia* (*phylum Verrucomicrobia*) and increased *Clostridium* and *Actinobacillus*. Additionally, a decreased presence of *Tenericutes* was found in breast cancer patients, which showed an association with higher severity of depressive symptoms (Bilenduke et al., 2022). Our results go in line with the association for *Verrucomicrobia* and *Tenericutes*. In breast cancer survivors, changes in anxiety were correlated with *Bacteroides* and *Coprococcus* (Paulsen et al., 2017), which in our sample were lower in the women who improved and worsened, respectively, in the anxiety subscale of the HADS. However, similar to our results, studies have shown that patients with generalized anxiety disorder, but not breast cancer, present a higher abundance of *Bacteroides* (Smith et al., 2022). Furthermore, while fewer depressive symptoms have been linked to lower Faecalibacterium levels, our participants with worse follow-up scores showed a reduction in this genus. Likewise, patients with major depressive disorder revealed an increase in *Roseburia* and *Bacteroides*. However, the latter was found to be reduced in the women who experienced worsened depressive symptoms in our sample (Paulsen et al., 2017). Due to the limited research found and the possible impact of other factors in the microbiota composition and the prevalence of anxiety and depression, the association between the two is not always linear.

This exploratory study did not investigate the mechanisms behind the relationship between microbiota composition and anxiety/depression. However, previous research indicates that individuals with these conditions often have lower levels of bacterial species that produce anti-inflammatory SCFAs, including butyrate and BDNF. Specifically, species such as *Faecalibacterium, Coprococcus, Clostridium* and *Bifidobacterium* are typically less abundant. SCFAs help regulate inflammation, cytokine profiles, and can cross the BBB, influencing brain mechanisms, potentially contributing to positive outcomes. Additionally, individuals with anxiety and depression often have fewer species involved in the production of neurotransmitters and their precursors, including serotonin, GABA, and tryptophan (Ciernikova et al., 2021; Simpson et al., 2021; Ortega et al., 2022). These mechanisms should be investigated more thoroughly in the breast cancer population.

Regarding the QoL assessment, most participants experienced a decline in their global scores. Inconsistencies found in literature likely stem from a lack of standardized methodology. A decrease of QoL in the course of chemotherapy, with physical and psychological deterioration, could be potentiated by treatments' demand and the emergence of side effects (Pitman et al., 2018; Lopes-Conceição et al., 2021; Zhang et al., 2018; Smith et al., 2022). Evidence revealed improved global scores over time, similar to our results, with worsening physical, role, cognitive, and social scores (Montazeri et al., 2008). The decline in physical scores, both before and during the first year of treatment (when it reveals the worst scores), as observed in our study at 3 months, is also reported in the literature (Lopes-Conceição et al., 2021; Montazeri et al., 2008; Moro-Valdezate et al., 2013). The evolution of our sample's symptom scale scores revealed mixed results, with a decline mostly found for fatigue. Evidence has shown that symptoms of anxiety, fear, and concerns regarding mortality may be present, as well as poor sleep, which can lead to mood disturbances and fatigue (Lopes-Conceição et al., 2021). Literature also revealed that most symptom scales worsen over time, except sleep (Montazeri et al., 2008). However, other results, in follow-up assessments later than 3 months, showed improvements in most symptom scales (Traore et al., 2018; Härtl et al., 2010).

Regarding the microbiotas' relative abundance, research in breast cancer survivors has found a correlation between higher levels of *Lachnospiraceae*, which belongs to the *Bacillota phylum*, and worst scores for the social functioning and mental health subscales of the Short Form Health-related QoL Survey-36 (SF-36). This differs from our results, likely due to different QoL screening tools and the fact that our participants had not yet started chemotherapy, which typically reduces this phylum's abundance (Montassier et al., 2015). However, similarly to our results, higher levels of *Ruminococcus* were associated with decreased physical functioning scales and mental health. This study also found a tendency for a negative association between *Faecalibacterium* with almost all subscales and *Bacteroides* with physical functioning and a positive association between *Prevotella* and physical functioning. This information aligns with our study (Smith et al., 2022). An association between increased fatigue levels, which is part of the symptom scale, and a higher abundance of *Prevotella* and *Faecalibacterium* has also been described in breast cancer survivors. This information differs from our results for the former (Ciernikova et al., 2021; Deleemans et al., 2022).

Since the evidence on the direct association between QoL and microbiota profile in breast cancer patients is limited, it was necessary to explore broader association. It has been demonstrated that proper diet can improve overall health, covering functional status and QoL (Amarantos et al., 2001). Higher adherence to the Mediterranean diet was associated with increased physical functioning and decreased pain scores and was revealed to positively impact QoL in breast cancer survivors (Porciello et al., 2020; Montagnese et al., 2020). Additionally, the Mediterranean diet is recognized to increase microbiota diversity, with a higher abundance of *Bacteroides, Bifidobacteria, Prevotella, Lactobacillus, Clostridium, Faecalibacterium, Ruminococcus, Oscillospira* and *Roseburia*, and a lower abundance of *Bacillota, Pseudomonadota* and *Coprococcus* (Jin et al., 2019; Beam et al., 2021). This suggests an association between the microbiota profile described and improved QoL. This evidence aligns with our results regarding higher abundances of *Bacteroides* and *Faecalibacterium* found in women who had improved QoL. However, a decreased Shannon index and increased *Bacillota* contradict this information. This inconsistency also occurs regarding the increased levels of *Prevotella* and *Ruminococcus* in patients with worsened QoL over time. Our sample presented a low adherence to the Mediterranean dietary pattern, which could have impacted their microbiota profile. In fact, our sample had a high relative abundance of *Bacillota* and *Pseudomonadota*, which are usually low in individuals who follow a Mediterranean diet (Jin et al., 2019). As mentioned before, this difference might also reflect chemotherapy's impact on microbiota, absent at the time of the microbiota profile assessment but likely present at the QoL's follow-up evaluation.

As previously described, breast cancer patients often present symptoms of anxiety, depression, and cognitive impairment. Evidence has found that QoL is associated with and can be impacted by these symptoms (Ciernikova et al., 2021; Lopes-Conceição et al., 2021; Valles-Colomer et al., 2019). The severity of depressive symptoms is a reliable predictor of global QoL among chemotherapy patients (Iconomou et al., 2004). The presence of negative emotions in breast cancer patients may have a significant impact on their psychological wellbeing, as well as their willingness to comply with treatment and engage in rehabilitation activities. Ultimately, this can result in a reduction of their overall QoL (Zhang et al., 2018). As stated earlier, evidence suggests that higher Shannon index and richness are associated with better cognitive function and reduced symptoms of anxiety and depression. This could be inferred from what would be found regarding QoL. Our QoL results align with this evidence regarding richness but not for alpha-diversity. As previously explained, even with a lower Shannon index found, the bacteria that thrived could have positively impacted patients' QoL, together with the possible positive effect of other factors. Regarding relative abundance, the literature shows that cognitive impairment, anxiety, and depression were associated with reduced abundance of *Verrucomicrobia* and augmented *Clostridium*. The associations were also made for less *Odoribacter, Bacillota, Ruminococcus, Lachnospira* and *Bacteroides* for cognitive impairment. The results of the present study are in line with these regarding the abundance of *Bacillota*. However, the opposite happens for *Bacteroides* and *Ruminococcus*. Nevertheless, direct comparisons are challenging due to differences in study protocols.

This study is novel in exploring how microbiota profile at the time of diagnosis may influence psychological symptoms during treatment. This new knowledge sheds light on potential strategies for modulating the microbiota before diagnosis, such as dietary interventions, which can promote a diverse and healthy microbiota that can withstand the impact of disease and treatments, ultimately enhancing the body's resilience throughout the entire process.

The present exploratory study revealed relevant insights; however, it also has some limitations worth mentioning. The sample size was relatively small, and due to logistical and time constraints, some data was missing at different timepoints. Nevertheless, it presented homogeneity, both in cancer type as well as in treatment scheme. Furthermore, variables beyond our control, such as race, menopausal status, mode of delivery, and breastfeeding, were similar among all participants. The questionnaires used were designed to assess overall anxiety, depression, cognitive function, and QoL. Thus, specific issues related to these side effects were not thoroughly examined. Likewise, while practical and efficient, these questionnaires are screening scales rather than diagnostic tools. Nevertheless, they are widely used in research on these topics. We also note that no correction for multiple testing was applied in the microbiome analysis due to the small sample size and exploratory nature of the study, warranting cautious interpretation of the results. This is an exploratory, prospective study, which was limited to the 3 month-mark after treatment initiation. Although modest, the results of the present study give us important preliminary information. The existing literature on this specific topic and population is limited, making this study both innovative and informative, as it sheds new light on an emerging issue frequently encountered in clinical practice. Additionally, this study further validates existing data on Portuguese breast cancer patients and opens up new possibilities for correlations and further research.

In summary, these preliminary results, of a topic which have not been extensively researched yet, showed relevant information, revealing that there is a tendency for altered microbiota profile to be associated with these symptoms, which gave us an insight into the possibility of using the microbiota to prevent their emergence and reduce their impact throughout the disease and the treatment. By advancing our understanding of these factors and their impact on breast cancer patients, clinicians can take a proactive approach to prevention and early intervention, ultimately improving patient outcomes (Ciernikova et al., 2021; Knuesel and Mohajeri, 2021; Ortega et al., 2022; Thu et al., 2023; Juan et al., 2022).

## Data Availability

The datasets presented in this study can be found in online repositories. The names of the repository/repositories and accession number(s) can be found in the article/Supplementary material.
