# Supplementary material for: Assessing mental health, cognitive function and quality of life of breast cancer patients: exploring associations with gut microbiota in an observational and preliminary study
Source: Front Psychol. 2026 Mar 11;17:1437697. doi: 10.3389/fpsyg.2026.1437697 (PMC13014481; doi:10.3389/fpsyg.2026.1437697)
Supplement: Supplementary file 1 [file Supplementary_file_1.pdf]

Supplementary Table S1. Cognitive Function scores at baseline and 3 months after.

Variable	N	T0	T1	p*
**MoCA**	7	24 [21-27]	24 [23-25]	0.343

Participants' median Montreal Cognitive Assessment (MoCA) score at baseline (T0) and 3-months (T1). n = 7. Values are median [interquartile range].

*Wilcoxon test according to normality. p-value for the comparison between T0 and T1 evaluations. Differences were considered significant if p < 0.05.

Supplementary Table S2. Anxiety and Depression scores at baseline and 3 months after.

Variable	N	T0	T1	p*
**HADS**	6	13 [4-14]	5 [4-9]	0.116
**HADS - Anxiety**	6	8 [4-10]	5 [1-6]	0.078
**HADS - Depression**	6	2 [1-3]	3 [2-5]	0.518

Participants' median Hospital Anxiety and Depression Scales (HADS) score at baseline (T0) and 3-months (T1). n = 6. Values are median [interquartile range].

*Wilcoxon test according to normality. Differences were considered significant if p < 0.05.

Supplementary Table S3. Quality-of-Life scores at baseline and 3 months after.

Variable	n	T0	T1	p*
**EORTC-QLQ-C30 - Global**	7	67 [58-83]	50 [42-67]	**0.034**
**EORTC-QLQ-C30 - Functioning scales**				
**Physical**	7	93 [87-100]	87 [80-87]	0.050
**Role**	6	100 [83-100]	83 [67-100]	0.414
**Emotional**	7	75 [75-92]	92 [83-92]	0.201
**Cognitive**	7	83 [83-89]	89 [83-94]	0.276
**Social**	7	100 [83-100]	100 [100-100]	1.000
**EORTC-QLQ-C30 - Symptom scales**				
**Fatigue**	7	11 [0-22]	33 [22-33]	**0.042**
**Nausea**	6	0 [0-0]	8 [0-17]	0.083
**Pain**	7	0 [0-17]	0 [0-17]	1.000
**Dyspnea**	7	0 [0-0]	0 [0-0]	1.000
**Insomnia**	7	33 [0-33]	33 [0-33]	0.783
**Appetite**	7	0 [0-0]	0 [0-33]	0.317
**Constipation**	7	0 [0-33]	0 [0-0]	0.317

**Diarrhea**	7	0 [0-0]	33 [0-33]	**0.046**
**Financial**	7	0 [0-0]	33 [0-33]	0.083

Participants' median European Organization for Research and Treatment of Cancer Quality-of-Life Questionnaire Core-30 (EORTC QLQ-C30) score at baseline (T0) and 3-months (T1). n = 6-7. Values are median [interquartile range].

*Wilcoxon test according to normality. p-value for the comparison between T0 and T1 evaluations. Differences were considered significant if $p < 0.05$. Bold data indicate statistically significant p-values.

Supplementary Table S4. Participants' microbiota profile for the Cognitive Function, Mental Health and QoL

**Evolution (T0 vs T1)**		**n**		**Alpha-diversity**	**Richness**
**MoCA**	**Improved**	3	7	2.58 ± 0.12	193 ± 11
	**Maintained**	1		2.69 [c]	212 [c]
	**Worsen**	3		2.43 ± 0.64	185 ± 31
**HADS**	**Improved**	5	6	2.47 ± 0.48	191 ± 23
	**Maintained**	0		-	-
	**Worsen**	1		2.49 [c]	181 [c]
**HADS - Anxiety**	**Improved**	4	6	2.35 ± 0.47	200 ± 11
	**Maintained**	1		2.94 [c]	153 [c]
	**Worsen**	1		2.49 [c]	181 [c]
**HADS - Depression**	**Improved**	3	6	2.65 ± 0.32	185 ± 28
	**Maintained**	0		-	-
	**Worsen**	3		2.30 ± 0.52	193 ± 17
**EORTC-QLQ-C30 - Global**	**Improved**	1	7	2.30 [c]	201 [c]
	**Maintained**	0		-	-
	**Worsen**	6		2.51 ± 0.42	188 ± 21
**Physical**	**Improved**	1	7	2.94 [c]	153 [c]
	**Maintained**	0		-	-
	**Worsen**	6		2.41 ± 0.37	196 ± 11
**Role**	**Improved**	2	6	2.51 ± 0.29	202 ± 1
	**Maintained**	1		1.71 [c]	186 [c]
	**Worsen**	3		2.71 ± 0.22	182 ± 30
**Emotional**	**Improved**	4	7	2.35 ± 0.47	200 ± 11
	**Maintained**	0		-	-
	**Worsen**	3		2.65 ± 0.25	177 ± 22
**Cognitive**	**Improved**	4	7	2.31 ± 0.44	196 ± 7
	**Maintained**	2		2.82 ± 0.17	183 ± 42
	**Worsen**	1		2.49 [c]	181 [c]
**Social**	**Improved**	2	7	2.51 ± 0.29	202 ± 1
	**Maintained**	4		2.47 ± 0.53	187 ± 25
	**Worsen**	1		2.49 [c]	181 [c]

**Fatigue**	**Improved**	0	7	-	-
	**Maintained**	2		2.70 ± 0.01	207 ± 7
	**Worsen**	5		2.39 ± 0.45	183 ± 19
**Nausea**	**Improved**	0	6	-	-
	**Maintained**	3		2.57 ± 0.23	205 ± 6
	**Worsen**	3		2.38 ± 0.62	173 ± 18
**Pain**	**Improved**	1	7	2.30 [c]	201 [c]
	**Maintained**	4		2.67 ± 0.20	183 ± 22
	**Worsen**	2		2.20 ± 0.70	199 ± 18
**Dyspnea**	**Improved**	0	7	-	-
	**Maintained**	2		2.48 ± 0.40	190 ± 19
	**Worsen**	0		-	-
**Insomnia**	**Improved**	2	7	2.62 ± 0.45	177 ± 34
	**Maintained**	2		2.64 ± 0.12	191 ± 7
	**Worsen**	3		2.13 ± 0.58	198 ± 16
**Appetite**	**Improved**	0	7	-	-
	**Maintained**	6		2.47 ± 0.43	189 ± 21
	**Worsen**	1		2.53 [c]	196 [c]
**Constipation**	**Improved**	3	7	2.71 ± 0.22	179 ± 25
	**Maintained**	3		2.51 ± 0.20	203 ± 8
	**Worsen**	1		1.71 [c]	186 [c]
**Diarrhea**	**Improved**	0	7	-	-
	**Maintained**	3		2.50 ± 0.21	195 ± 12
	**Worsen**	4		2.47 ± 0.53	187 ± 25
**Financial difficulties**	**Improved**	0	7	-	-
	**Maintained**	4		2.47 ± 0.53	187 ± 25
	**Worsen**	3		2.50 ± 0.21	195 ± 12

Participants' Shannon index and richness for the Montreal Cognitive Assessment (MoCA), Hospital Anxiety and Depression Scales (HADS) and European Organisation for Research and Treatment of Cancer Quality of Life Questionnaire Core-30 (EORTC QLQ-C30) scores evolution from baseline (T0) to 3-months (T1) (improved, maintained or worsen in accordance with the respective questionnaire's scoring system). n = 6-7. Values are mean ± standard deviation (SD).

[c] No SD found, as only one participant fell into this category
